# Knockdown of lncRNA XR_877193.1 suppresses ferroptosis and promotes osteogenic differentiation via the PI3K/AKT signaling pathway in SONFH: LncRNA XR_877193.1 knockdown Inhibits ferroptosis of MC3T3-E1 cells

**DOI:** 10.3724/abbs.2025014

**Published:** 2025-03-14

**Authors:** Huixia Yang, Ning Ding, Shi Qing, Yinju Hao, Cilin Zhao, Kai Wu, Guizhong Li, Huiping Zhang, Shengchao Ma, Zhigang Bai, Yideng Jiang

**Affiliations:** 1 NHC Key Laboratory of Metabolic Cardiovascular Diseases Research Ningxia Medical University Yinchuan 750004 China; 2 Department of Medical Genetics Maternal and Child Health of Hunan Province Changsha 410008 China; 3 Ningxia Key Laboratory of Vascular Injury and Repair Research Ningxia Medical University Yinchuan 750004 China; 4 School of Basic Medical Sciences Ningxia Medical University Yinchuan 750004 China; 5 Department of Orthopedics People’s Hospital of Ningxia Hui Autonomous Region Yinchuan 750004 China; 6 General Hospital of Ningxia Medical University Yinchuan 750004 China

**Keywords:** steroid-induced osteonecrosis of the femoral head, ferroptosis, lncRNA XR_877193.1, PI3K/AKT signaling pathway

## Abstract

Ferroptosis is a novel form of regulated cell death characterized by the iron-dependent accumulation of lipid peroxides. Recent research has suggested that ferroptosis in osteoblasts contributes to steroid-induced osteonecrosis of the femoral head (SONFH). However, the relationship between ferroptosis and SONFH remains unclear. In this study, *in vitro* experiments show that dexamethasone (Dex) treatment reduces the expressions of key ferroptosis regulators, SLC7A11 and GPX4, in MC3T3-E1 cells. This reduction leads to a decrease in intracellular glutathione (GSH) levels, accompanied by elevated levels of total iron, malondialdehyde (MDA), and reactive oxygen species (ROS). Importantly, the ferroptosis inhibitor ferrostatin-1 (Fer-1) effectively reverses Dex-induced ferroptosis in MC3T3-E1 cells. Furthermore, RNA-seq analysis reveals that the long noncoding RNA (lncRNA) XR_877193.1is significantly upregulated in Dex-treated MC3T3-E1 cells. Functional studies demonstrate that the knockdown of lncRNA XR_877193.1 promotes osteogenic differentiation by inhibiting Dex-induced ferroptosis in MC3T3-E1 cells, whereas its overexpression exacerbates cell death via ferroptosis. Kyoto Encyclopedia of Genes and Genomes (KEGG) enrichment analysis reveals that the differentially expressed lncRNA XR_877193.1 is enriched in ferroptosis-related pathways, including the PI3K/AKT signaling pathway. Moreover, PI3K/AKT inhibitors reverse ferroptosis in MC3T3-E1 cells inhibited by lncRNA XR_877193.1 knockdown. Collectively, our findings indicate that lncRNA XR_877193.1 knockdown exerts anti-ferroptosis effects by stimulating the PI3K/AKT signaling pathway, suggesting a promising therapeutic strategy for attenuating SONFH.

## Introduction

Glucocorticoids (GCs) are widely used for the treatment of inflammatory and autoimmune diseases or cancers, including arthritis, systemic lupus erythematous and relapsed or refractory multiple myeloma [ 1, 2]. However, in recent years, numerous studies have demonstrated that excessive GC usage inhibits osteoblast proliferation and promotes their death, thus resulting in the occurrence of steroid-induced osteonecrosis of the femoral head (SONFH) in patients [3]. With the widespread misuse of glucocorticoids, the incidence of this disease has been steadily increasing [4]. Clinically, this condition primarily manifests as hip pain, restricted hip joint mobility, and, in severe cases, may lead to bone collapse [5]. Therefore, early diagnosis and intervention are crucial for preventing the progression of this disease.

Osteoblasts are key cells in bone formation. GCs reduce the proliferation and differentiation of osteoblast precursor cells by downregulating key markers such as Runx2, Osterix, and ALP [6]. GCs also induce osteoblast death, diminishing their overall numbers and further reducing bone formation over time [ 7, 8]. Additionally, GC exposure decreases ALP activity, impairing mineralization of the bone matrix and resulting in weaker, less dense bone [9]. Although the primary effect of GCs is on osteoblasts, they also indirectly increase osteoclast activity. This occurs through the upregulation of RANKL expression in osteoblasts and a reduction in osteoprotegerin, which normally inhibits RANKL. Increased osteoclast activity accelerates bone resorption, compounding the negative effects on bone formation and contributing to osteoporosis and SONFH [10]. Understanding these mechanisms may reveal potential therapeutic targets for SONFH.

Recent studies have indicated that ferroptosis significantly contributes to the pathogenesis of SONFH [11]. GC treatment induces oxidative stress and disrupts iron homeostasis, resulting in lipid peroxidation and ferroptosis in osteocytes and osteoblasts. The inhibition of system XC-, which transports cystine and affects GSH synthesis, lowers GSH levels and exacerbates oxidative stress, further promoting ferroptosis in bone cells [12]. It has also been reported that increased iron accumulation in bone tissue can drive lipid peroxidation and activate ferroptosis [13]. Novel therapeutic strategies that target ferroptosis, such as ferroptosis inhibitors and agents that restore GSH levels, are under investigation to protect bone cells from oxidative damage and improve bone health in SONFH patients [14]. Studying the molecular mechanisms of ferroptosis and its potential as a therapeutic target will provide hope for improving therapeutic strategies for SONFH.

In recent years, with the development of high-throughput sequencing technology, abnormal gene expression and epigenetic changes have become the focus of disease pathogenesis research. In particular, in recent years, most studies have focused on the molecular regulatory mechanism of noncoding RNAs (ncRNAs) in diseases. Among them, long noncoding RNAs (lncRNAs) are RNA transcripts that are longer than 200 nt and exhibit limited or no protein-coding capacity [15]. Recent evidence has revealed that lncRNAs, key regulatory molecules with multiple biological functions, can regulate different signaling pathways through various mechanisms to participate in biological processes such as differentiation, proliferation, and apoptosis, thus affecting the process of disease [16]. Recently, some studies revealed that aberrant expression of lncRNAs is associated with SONFH. For example, lncRNA FAR591 is highly expressed in SONFH, and knockout of lncRNA FAR591 can effectively block glucocorticoid-induced apoptosis of BMECs, thereby inhibiting the occurrence and progression of SONFH [17]. However, current research on the regulation of SONFH by lncRNAs is still limited, and lncRNAs with potential regulatory roles in SONFH remain to be discovered.

In the present study, we examined the expression profiles of lncRNAs in Dex-treated MC3T3-E1 cells. The lncRNA XR_877193.1 was considerably upregulated in SONFH and was linked to MC3T3-E1 cell ferroptosis. Research has shown that the lncRNA XR_877193.1 might regulate MC3T3-E1 cell ferroptosis via the PI3K/AKT signaling pathway, which provides a new target for the diagnosis and prognosis of SONFH.

## Materials and Methods

### Cell culture

Mouse MC3T3-E1 cells were purchased from Zhongqiao Xinzhou (Shanghai, China). The cells were cultured in MEMα (Zhongqiao Xinzhou) supplemented with 10% fetal bovine serum (Zhongqiao Xinzhou) and 1% penicillin-streptomycin (Solarbio, Beijing, China) in a 5% CO _2_ atmosphere.

### Cell treatment

MC3T3-E1 cells were treated with different doses of Dex (0.1, 0.5, 1 and 5 μM; Sigma, St Louis, USA) or LY294002 (1.25, 2.5, 5, 10, 20, and 40 μM; Abmole, Shanghai, China) for screening the optimum concentration. Then, MC3T3-E1 cells were treated with 1 μM Dex in the absence or presence of 10 μM ferrostatin-1 (Fer-1, a ferroptosis inhibitor; MCE, Monmouth Junction, USA). MC3T3-E1 cells without treatment were used as control.

### Cell transfection

MC3T3-E1 cells (3 × 10 ^3^ cells/well) were cultured in 6-well plates, and siRNAs targeting lncRNA XR_877193.1, the lncRNA XR_877193.1 overexpression plasmid and the corresponding control vectors constructed by GenePharma Company (Shanghai, China) were transfected into MC3T3-E1 cells using Lipofectamine ^TM^ 2000 (Invitrogen, Carlsbad, USA) according to standard protocols. At 24 h post-transfection, the cells were harvested for further experiments. The siRNA sequences of lncRNA XR_877193.1 are listed in Supplementary Table S1.

### Biochemical detection

The concentrations of total iron, malondialdehyde, and glutathione were measured using the corresponding kits (NJJCBIO, Nanjing, China) according to the manufacturer’s instructions.

### Quantitative real-time polymerase chain reaction (qRT-PCR) analysis

The levels of lncRNA XR_877193.1, GPX4 and SLC7A11 were analyzed by qRT-PCR. Total RNA was isolated using a phenol-chloroform extraction kit (TIANGEN, Beijing, China) following the manufacturer’s instructions. Single-strand cDNA was synthesized using a SuperScript™ IV One-Step qRT-PCR System (Thermo Fisher Scientific, Waltham, USA). qRT-PCR was conducted with SGExcel FastSYBR Mixture (Sangon, Shanghai, China) on a QuantStudio™ RT-PCR System (Thermo Fisher Scientific). The fluorescence signal at the end of each amplification cycle was measured to monitor the accumulation of PCR products and determine Ct values for each sample. Ct values were normalized to *GAPDH* to correct for variability in RNA input and RT efficiency. The relative mRNA expression levels were quantified between sample groups using the 2 ^‒ΔΔCt^ method. The primer sequences are shown in Supplementary Table S2.

### Western blot analysis

The proteins were separated by 10% SDS-PAGE and transferred onto PVDF membranes (Millipore, Billerica, USA). The samples were subsequently incubated in TBST buffer with 5% nonfat milk for 2 h at room temperature. The membranes were incubated overnight at 4°C with the following primary antibodies: anti-GPX4 (1:1000; Abcam, Cambridge, UK), anti-SLC7A11 (1:1000; Abcam), anti-p-PI3K (1:1000; Affinity, Nanjing, China), anti-PI3K (1:1000; Affinity), anti-p-AKT (1:1000; Affinity), anti-AKT (1:1000; Affinity), and anti-β-actin (1:3000; Santa Cruz Biotech, Santa Cruz, USA). The membranes were washed again and incubated with HRP-conjugated goat anti-rabbit (H+L) or goat anti-mouse (H+L) secondary antibody (1:5000; ZSGB-BIO, Beijing, China). The target proteins were detected using chemiluminescence reagent. Finally, the intensity of the protein bands on the membrane was quantified via Image Lab analysis software, allowing for the comparison of protein expression levels between different samples.

### Transmission electron microscopy (TEM)

MC3T3-E1 cells were fixed with 1% pentanoic acid prepared with 0.1 M phosphate buffer (pH 7.4) for 2 h, dehydrated with alcohol and acetone, and then permeated with acetone and embedding agent. After being embedded, the cells were cut into 60–80-nm ultrathin slices and stained with 2% uranium acetate saturated alcohol and 2.6% lead citrate. MC3T3-E1 cells were observed with a transmission electron microscope (Hitachi, Tokyo, Japan), and images were collected for analysis.

### Immunofluorescence staining

MC3T3-E1 cells were cultured on coverslips until they reached confluence. MC3T3-E1 cells were treated with Dex for 24 h before being fixed, permeabilized, and incubated with rabbit anti-mouse GPX4 and SLC7A11 antibodies (Abcam). After being stained with goat anti-rabbit lgG (H+L) Fluor 647- or goat anti-rabbit lgG (H+L) Fluor-488-conjugated (Affinity), the cells were counterstained with DAPI. Images were acquired using a confocal laser scanning microscope (ZEISS, Wetzlar, Germany), and the fluorescence intensity of the cells was assessed by imaging.

### Microarray-based lncRNA profiling

Total RNA was extracted from 3 pairs of normal and Dex-treated MC3T3-E1 cells. RNA-seq was carried out by Shanghai Ouyi Biomedical Technology Co., Ltd (Shanghai, China), and the original data collected were analyzed and subsequently processed using Ouyi Cloud software. Differentially expressed lncRNAs were screened by |log2FC| ≥ 2 and *P* < 0.05. Moreover, gene ontology (GO) and Kyoto Encyclopedia of Genes and Genomes (KEGG) analyses were performed on the target genes of the differentially expressed lncRNAs to better understand their functional significance.

### Coding potential assessment tool (CPAT)

The CPAT ( http://lilab.research.bcm.edu/) is commonly used to predict the coding potential of RNA sequences, particularly for distinguishing long non-coding RNAs (lncRNAs) from protein-coding RNAs. To use CPAT, the lncRNA XR_877193.1 sequences in FASTA format were collected and processed to extract the open reading frames (ORFs). These sequences were then analyzed with CPAT using the command line interface. By using this tool, we analyzed features like ORF length and nucleotide composition, assigning a score between 0 and 1. Sequences with lower scores (typically < 0.5) were classified as non-coding, suggesting they are lncRNAs.

### RNA fluorescence *in situ* hybridization (RNA-FISH)

The lncRNA XR_877193.1 probe and fluorescence *in situ* hybridization kit purchased from GenePharma (Shanghai, China) were used for RNA-FISH to identify the location and expression of lncRNA XR_877193.1 in MC3T3-E1 cells. Hybridization conditions and imaging were performed according to the instructions provided in the FISH manual (GenePharma). The lncRNA XR_877193.1 probe sequence was as follows: 5′-TCTGACCTCCGTTCACATCTTG-3′.

### RNA isolation from nuclear and cytoplasmic fractions

The RNA isolation procedure employs spin column chromatography with Norgen’s proprietary resin (Norgen, Thorold, Canada). The cells were first lysed with lysis buffer. After lysis, the mixture was centrifuged to separate the cytoplasmic RNA in the supernatant from the nuclear RNA in the pellet. Each fraction was then mixed with buffer SK and ethanol. The RNA-bound column was washed with wash solution A to eliminate residual impurities. Finally, the purified RNA was eluted with elution buffer E. The resulting total RNA samples from both the nuclear and the cytoplasmic fractions were then subjected to qRT-PCR analysis.

### Alizarin red staining

To evaluate the osteogenic differentiation of MC3T3-E1 cells, third-generation MC3T3-E1 cells were seeded into 6-well plates. After different treatments, the culture medium was discarded, and the cells were washed twice with PBS, fixed in 4% paraformaldehyde for 15 min, and stained with Alizarin Red reagent (Servicebio, Wuhan, China). Microscopic observation was then conducted to assess mineralized matrix formation.

### 2′,7′-Dichlorodihydrofluorescein diacetate (DCFH-DA) staining

Intracellular reactive oxygen species (ROS) levels were evaluated using DCFH-DA staining kit (KeyGEN, Nanjing, China). MC3T3-E1 cells were seeded in confocal dishes and subjected to various treatments. Following treatment, adherent cells were rinsed with PBS three times and incubated with 10 μM DCFH-DA for 30 min at 37°C in the dark. The average fluorescence intensity of each group was measured with a confocal laser scanning microscope to determine the intracellular ROS levels, and the fluorescence intensity was quantified with ImageJ.

### Flow cytometry analysis of intracellular ROS

DCFH-DA can be hydrolyzed to DCFH after crossing the cell membrane, and ROS in the cell can oxidize nonfluorescent DCFH to produce fluorescent DCF. The cells were collected and stained with DCFH-DA (KeyGEN) for 20 min according to the manufacturer’s instructions. Flow cytometry was used to detect the fluorescence of DCF to determine the level of intracellular ROS.

### CCK-8 assay

MC3T3-E1 cell suspensions were inoculated into a 96-well plate. After pre-culturing for 24 h at 37°C with 5% CO _2_, different concentrations of test substances were added to each well. After 24 h of treatment, 10 μL of CCK-8 reagent (LABLEAD, Beijing, China) was added to each well and gently mixed. The plate was incubated for an additional 2 h. The absorbance (OD) at 450 nm was then measured using a microplate reader (Epoch; BioTek, Winooski, USA), and the data were recorded for analysis.

### Statistical analysis

The experimental data were analyzed using the statistical software GraphPad Prism 8.0. Data are presented as the mean ± standard deviations (SD). The two groups were compared using Student’s *t* test. ANOVA followed by the Newman-Keuls test was used to compare multiple groups. *P < *0.05 was considered statistically significant.

## Results

### Dex promotes ferroptosis in MC3T3-E1 cells

**Figure 1:**
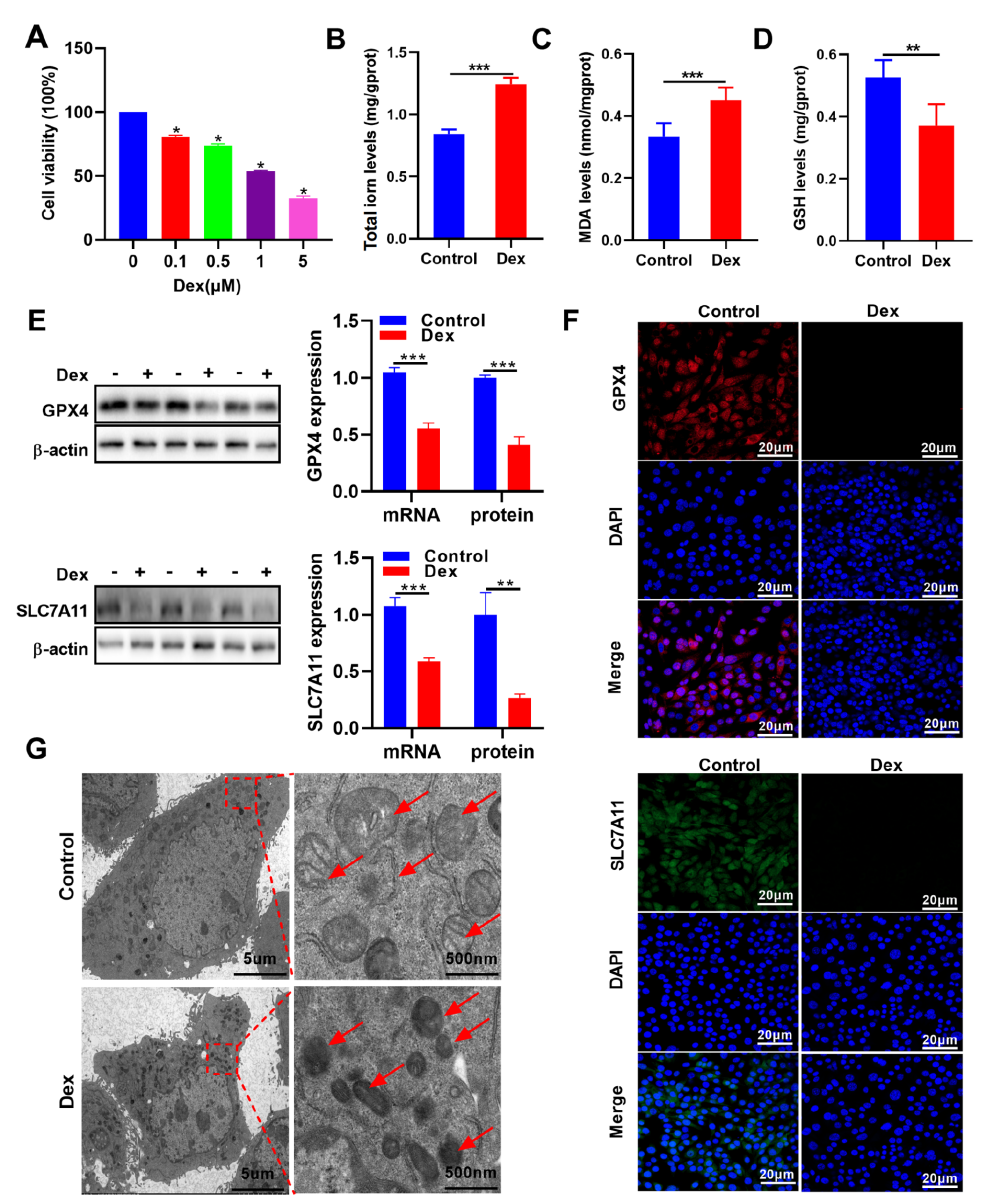
Figure 1 Dex promotes ferroptosis in MC3T3-E1 cells (A) The CCK-8 assay results of MC3T3-E1 cells treated with different concentration of Dex (n = 3). (B–D) The concentrations of total iron, MDA, and GSH were measured using biochemical kits in MC3T3-E1 cells treated with Dex (n = 6). (E) The mRNA and protein expression levels of GPX4 and SLC7A11 were measured by qRT-PCR and western blot analysis in MC3T3-E1 cells treated with Dex (n = 3). (F) Immunofluorescence staining of GPX4 (red) and SLC7A11 (green) in MC3T3-E1 cells treated with Dex. The nuclei were stained with DAPI (blue). Scale bar: 20 μm (G) Transmission electron microscopy (TEM) images showing changes in mitochondria in MC3T3-E1 cells treated with Dex. Scale bar: 5 μm (left). Scale bar: 500 nm (right). Data are presented as the mean ± SD. *P < 0.05, **P < 0.01, ***P < 0.001.

To explore the role of Dex in MC3T3-E1 cells, MC3T3-E1 cells were treated with different concentrations of Dex for 24 h to establish an *in vitro* model of SONFH. After 24 h of Dex treatment, the CCK-8 assay results revealed a significant decrease in cell viability, with an estimated IC _50_ value of approximately 1 μM ( Figure 1A). Thus, we used a treatment with 1 μM Dex for all subsequent cell experiments. Moreover, this model has already been reported by others [18]. We subsequently assessed iron accumulation and lipid peroxidation, which are critical signaling events in triggering ferroptosis. The results indicated that both the total iron and MDA concentrations were notably higher in the Dex group than in the control group, whereas the level of GSH was significantly decreased in the Dex group ( Figure 1B–D). Additionally, qRT-PCR and western blot analysis were used to measure the expression levels of ferroptosis-related proteins. The results revealed that the expression levels of GPX4 and SLC7A11 were significantly lower in the Dex group than in the control group ( Figure 1E). These changes were further confirmed by immunofluorescence staining ( Figure 1F). Moreover, transmission electron microscopy revealed structural changes in the mitochondria of MC3T3-E1 cells following Dex treatment. Specifically, we observed that the number of mitochondria decreased and that the number of mitochondrial ridges decreased significantly compared with those in the control group ( Figure 1G). Collectively, these findings suggest that Dex promotes ferroptosis in MC3T3-E1 cells, highlighting its potential role in the pathophysiology of SONFH.

### Fer-1 promotes osteogenic differentiation by inhibiting Dex-induced ferroptosis in MC3T3-E1 cells

**Figure 2:**
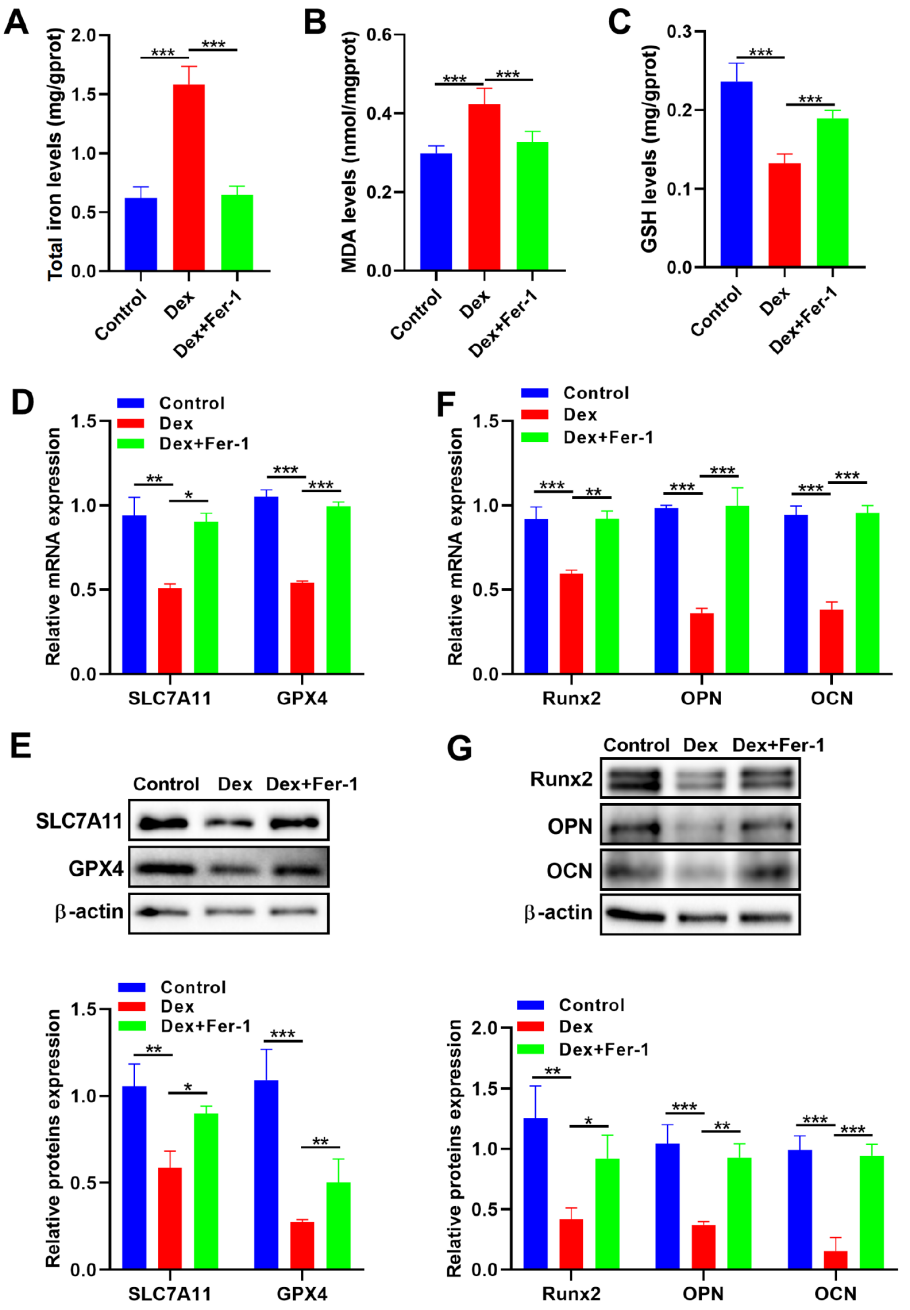
Figure 2 Fer-1 promotes osteogenic differentiation by inhibiting Dex-induced ferroptosis in MC3T3-E1 cells (A–C) The concentrations of total iron, MDA, and GSH were detected using biochemical kits in MC3T3-E1 cells treated with Dex and Fer-1 (n = 6). (D,E) The mRNA and protein expression levels of GPX4 and SLC7A11 were measured by qRT-PCR and western blot analysis in MC3T3-E1 cells treated with Dex and Fer-1 (n = 3). (F,G) The mRNA and protein expression levels of Runx2, OPN and OCN were measured by qRT-PCR and western blot analysis in MC3T3-E1 cells treated with Dex and Fer-1 (n = 3). Data are presented as the mean ± SD. *P < 0.05, **P < 0.01, ***P < 0.001.

To confirm the role of ferroptosis in Dex-induced SONFH *in vitro*, MC3T3-E1 cells were treated with 10 μM ferrostatin-1 (Fer-1, a ferroptosis inhibitor) or 1 μM Dex. The total intracellular iron and MDA levels significantly increased under Dex stimulation but decreased following Fer-1 treatment. Moreover, GSH levels significantly increased ( Figure 2A–C). After Fer-1 treatment, the expressions of GPX4 and SLC7A11 were higher than those in the Dex group ( Figure 2D,E). We subsequently explored the effect of Fer-1 on Dex-induced osteogenic differentiation of MC3T3-E1 cells. Fer-1 treatment increased the expressions of osteogenic differentiation markers, including OPN, runt-related transcription factor 2 (Runx2), and osteocalcin (OCN) ( Figure 2F,G). Collectively, these results indicate that Fer-1 alleviates osteogenic differentiation by inhibiting Dex-induced ferroptosis in MC3T3-E1 cells.

### LncRNA XR_877193.1 is upregulated in Dex-treated MC3T3-E1 cells

**Figure 3:**
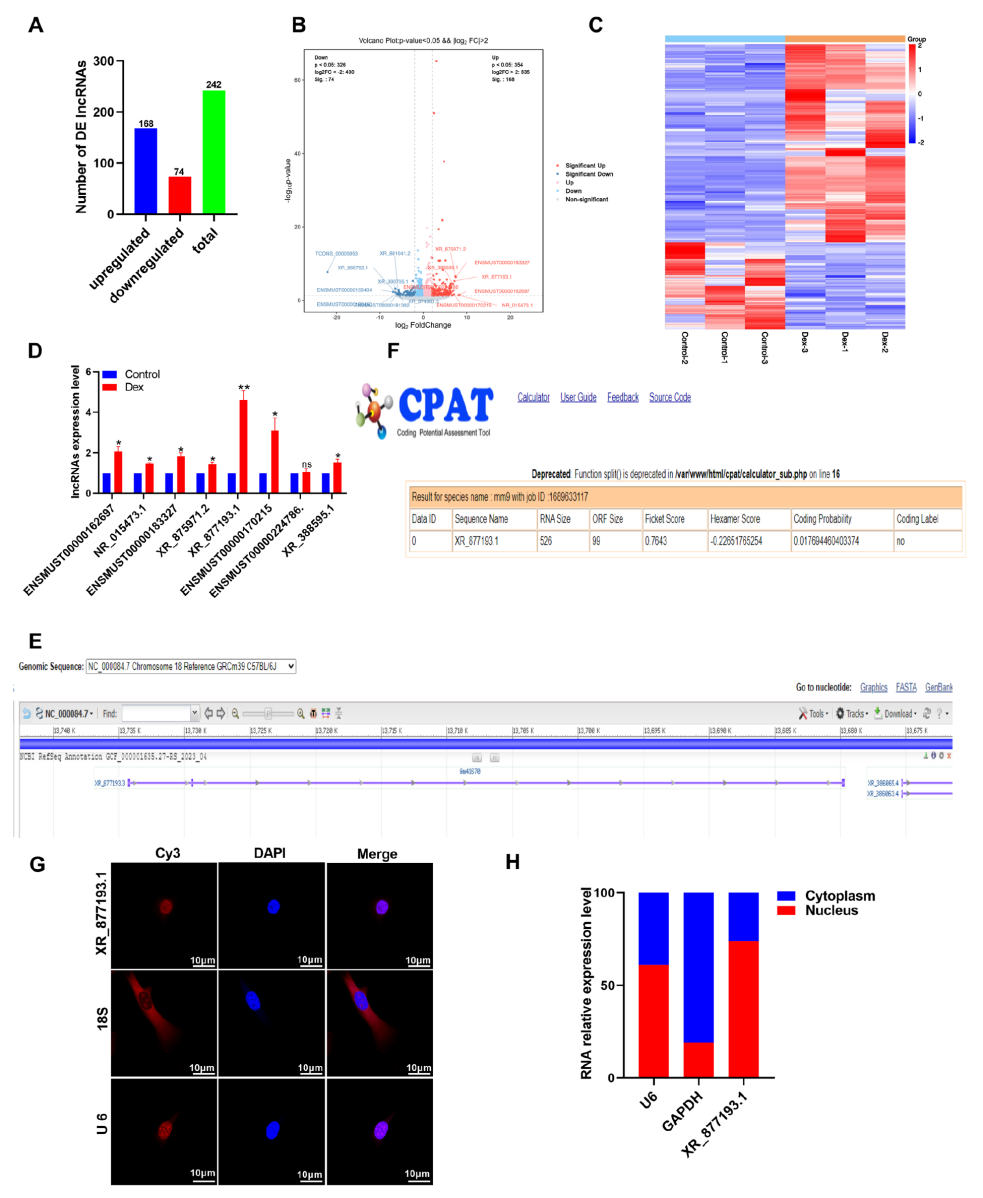
Figure 3 Identification of lncRNA XR_877193.1 in Dex-treated MC3T3-E1 cells (A) The number of differentially expressed lncRNAs in Dex-treated MC3T3-E1 cells was presented. (B) Volcano plots of differentially expressed lncRNAs in Dex-treated MC3T3-E1 cells were generated. Red dots indicate significant upregulation of lncRNAs, blue dots indicate significant downregulation of lncRNAs and grey dots represent no difference. (C) A cluster heatmap showing the significantly dysregulated lncRNAs in Dex-treated MC3T3-E1 cells. (D) qRT-PCR analysis of the 8 most upregulated lncRNAs in Dex-treated MC3T3-E1 cells (n = 3). (E) National Center for Biotechnology Information (https://www.ncbi.nlm.nih.gov/) was used to search the location and conservativeness of lncRNA XR_877193.1 (NC-000084.7 Chromosome 18 Reference GRCm39 C57BL/6J). (F) The CPAT (http://lilab.research.bcm.edu/) predicted the protein-coding power of lncRNA XR_877193.1. (G) RNA-FISH assay was used to detect the subcellular location of lncRNA XR_877193.1 in MC3T3-E1 cells. The nuclei were stained with DAPI (blue). Scale bar: 10 μm. (H) lncRNA XR_877193.1 expression was analyzed by PCR in the nuclei and cytoplasmic extracts of MC3T3-E1 cells. The cytoplasmic mRNA control was GAPDH, and the nuclear RNA control was U6. Data are presented as the mean ± SD. *P < 0.05, **P < 0.01.

To identify the key lncRNAs involved in SONFH progression, we utilized RNA-seq to examine changes in lncRNA expression in MC3T3-E1 cells treated with Dex. A total of 242 differentially expressed lncRNAs, including 168 upregulated and 74 downregulated lncRNAs ( Figure 3A–C), were identified on the basis of the criteria of |log2FC| ≥ 2 and *P*  < 0.05. To validate the RNA-seq data, we selected the 8 most upregulated lncRNAs and confirmed their expressions by qRT-PCR in MC3T3-E1 cells. The results revealed that 7 of these genes were significantly upregulated in MC3T3-E1 cells treated with Dex, with lncRNA XR_877193.1 exhibiting the highest expression ( Figure 3D). Consequently, lncRNA XR_877193.1 was selected for subsequent functional studies. Bioinformatics analysis revealed that lncRNA XR_877193.1 is located on C57BL/6J chromosome 18 ( Figure 3E). The Coding Potential Assessment Tool (CPAT) confirmed that lncRNA XR_877193.1 is a noncoding RNA lacking protein-coding capacity ( Figure 3F). Furthermore, RNA-FISH and nuclear/cytosol fractionation assays revealed that lncRNA XR_877193.1 was distributed mainly in the nucleus of MC3T3-E1 cells ( Figure 3G,H). These findings suggest that lncRNA XR_877193.1 may play a crucial role in SONFH.

### LncRNA XR_877193.1 inhibits Dex-induced osteogenic differentiation of MC3T3-E1 cells

**Figure 4:**
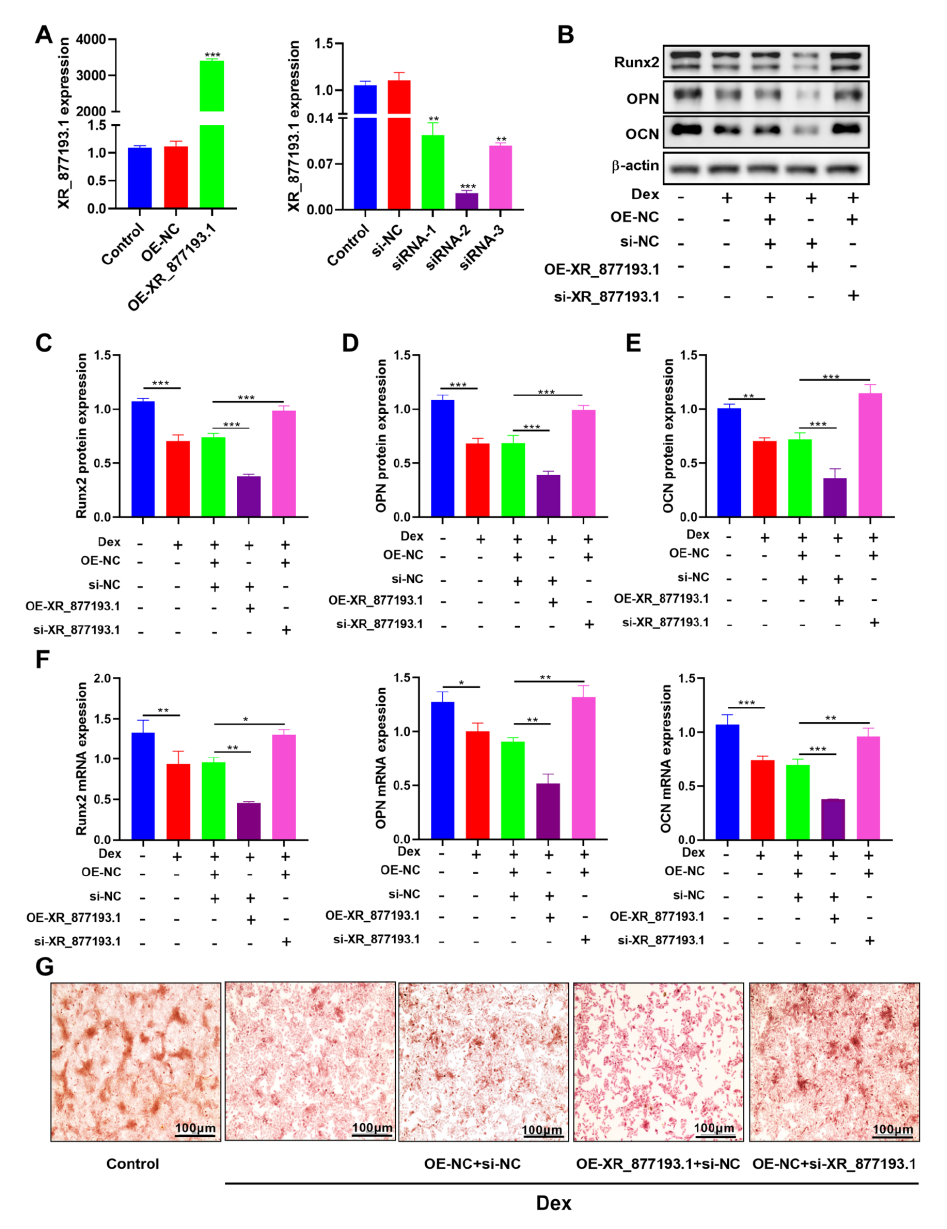
Figure 4 LncRNA XR_877193.1 inhibits Dex-induced osteogenic differentiation of MC3T3-E1 cells (A) The expression of lncRNA XR_877193.1 was measured by qRT-PCR in MC3T3-E1 cells transfected with lncRNA XR_877193.1 overexpression plasmid or lncRNA XR_877193.1 siRNAs (n = 3). (B–E) The expressions of Runx2, OPN and OCN were detected by western blot analysis in MC3T3-E1 cells transfected with lncRNA XR_877193.1 overexpression plasmid or lncRNA XR_877193.1 siRNA in the presence of Dex (n = 3). (F) The expressions of Runx2, OPN and OCN were measured by qRT-PCR in MC3T3-E1 cells transfected with lncRNA XR_877193.1 overexpression plasmid or lncRNA XR_877193.1 siRNA in the presence of Dex (n = 3). (G) Alizarin red staining of MC3T3-E1 cells transfected with lncRNA XR_877193.1 overexpression plasmid or lncRNA XR_877193.1 siRNA in the presence of Dex. Scale bar: 100 μm. Data are presented as the mean ± SD. *P < 0.01, **P < 0.01, ***P < 0.001.

To investigate the effect of lncRNA XR_877193.1 on osteogenic differentiation, MC3T3-E1 cells were transfected with the lncRNA XR_877193.1 overexpression plasmid or lncRNA XR_877193.1 siRNAs. As shown in Figure 4A, the qRT-PCR results revealed that the cells transfected with the lncRNA XR_877193.1 overexpression plasmid presented upregulated expression of lncRNA XR_877193.1. In contrast, the cells transfected with three different lncRNA XR_877193.1 siRNAs presented reduced expression of lncRNA XR_877193.1. Among them, lncRNA XR_877193.1 siRNA-2 had the strongest interference effect and was therefore selected for subsequent experimental research. The results demonstrated that the overexpression of lncRNA XR_877193.1 decreased the formation of calcified nodules and reduced the expression of osteogenic markers in Dex-treated MC3T3-E1 cells. In contrast, the knockdown of lncRNA XR_877193.1 resulted in a significant increase in calcified nodule formation and increased the expression of osteogenic markers ( Figure 4B–G). Taken together, these results indicate that lncRNA XR_877193.1 inhibits Dex-induced osteogenic differentiation of MC3T3-E1 cells.

### LncRNA XR_877193.1 promotes Dex-induced ferroptosis in MC3T3-E1 cells

**Figure 5:**
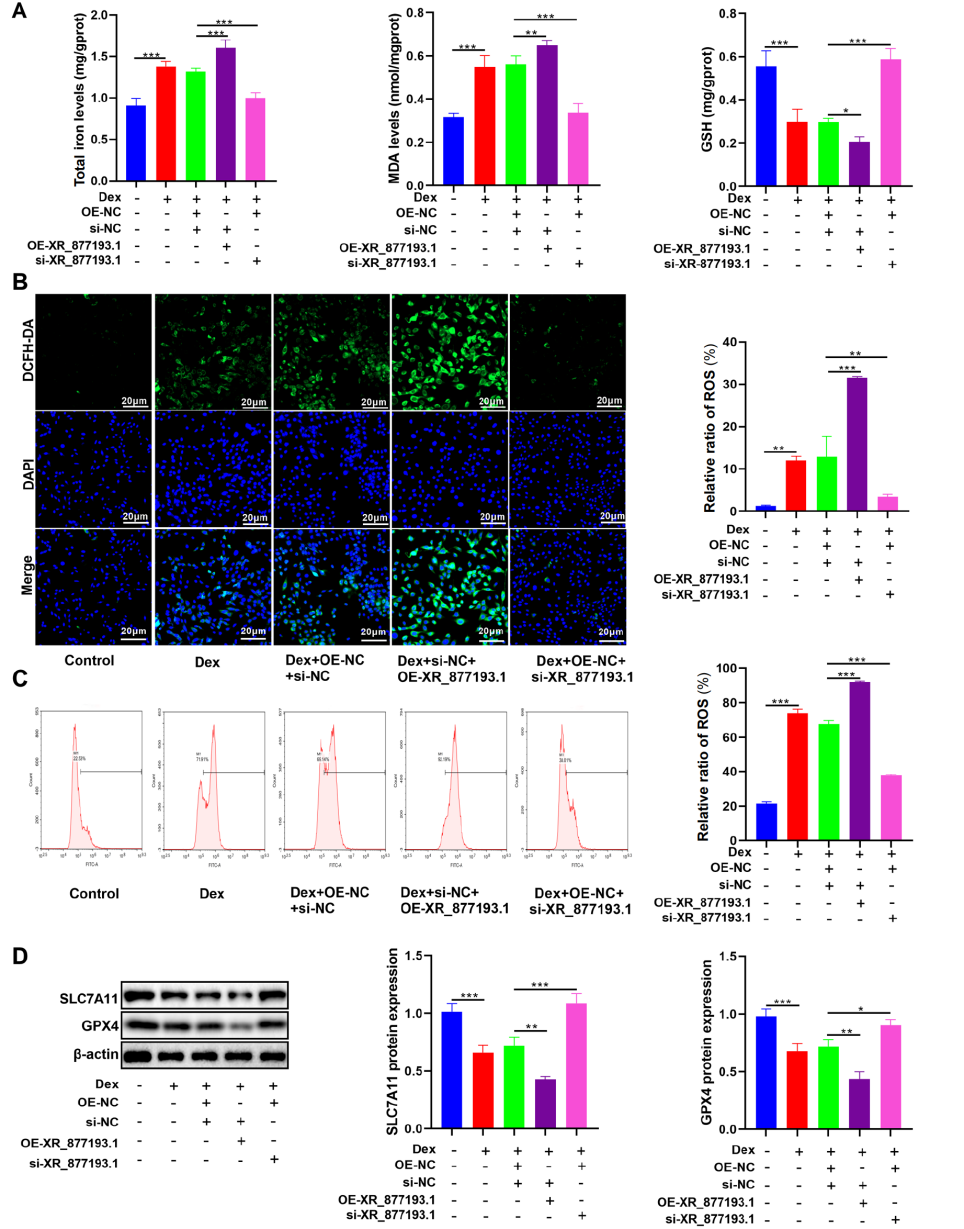
Figure 5 LncRNA XR_877193.1 promotes Dex-induced ferroptosis in MC3T3-E1 cells (A) The concentrations of total iron, MDA and GSH were detected using biochemical kits in MC3T3-E1 cells transfected with lncRNA XR_877193.1 overexpression plasmid or lncRNA XR_877193.1 siRNA in the presence of Dex (n = 6). (B) DCFH-DA staining was performed to evaluate intracellular ROS levels in MC3T3-E1 cells transfected with lncRNA XR_877193.1 overexpression plasmid or lncRNA XR_877193.1 siRNA in the presence of Dex (n = 3). The nuclei were stained with DAPI (blue). Scale bar: 20 μm. (C) Flow cytometry was performed to evaluate intracellular ROS levels in MC3T3-E1 cells transfected with lncRNA XR_877193.1 overexpression plasmid or lncRNA XR_877193.1 siRNA in the presence of Dex (n = 3). (D) Western blot analysis of SLC7A11 and GPX4 expressions in MC3T3-E1 cells transfected with lncRNA XR_877193.1 overexpression plasmid or lncRNA XR_877193.1 siRNA in the presence of Dex (n = 3). Data are presented as the mean ± SD. *P < 0.05, **P < 0.01, ***P < 0.001.

We subsequently examined the effect of lncRNA XR_877193.1 on ferroptosis in Dex-treated MC3T3-E1 cells. Our results revealed that the overexpression of lncRNA XR_877193.1 led to significant increases in total iron and MDA concentrations, whereas GSH levels were markedly reduced. In contrast, downregulation of lncRNA XR_877193.1 expression resulted in decreased levels of total iron and MDA, along with a significant increase in the GSH concentration in Dex-treated MC3T3-E1 cells ( Figure 5A). Next, we assessed intracellular ROS levels using DCFH-DA fluorescence staining and flow cytometry. As shown in Figure 5B,C, overexpression of lncRNA XR_877193.1 increased the ROS level in Dex-treated MC3T3-E1 cells, whereas the silencing of lncRNA XR_877193.1 significantly reduced the ROS level. Additionally, overexpression of lncRNA XR_877193.1 resulted in a decrease in GPX4 and SLC7A11 expression in Dex-treated MC3T3-E1 cells. Conversely, knockdown of lncRNA XR_877193.1 led to the opposite effect ( Figure 5D). In summary, our findings suggest that the lncRNA XR_877193.1 promotes Dex-induced ferroptosis in MC3T3-E1 cells.

### LncRNA XR_877193.1 suppresses the PI3K/AKT signaling pathway in MC3T3-E1 cells

**Figure 6:**
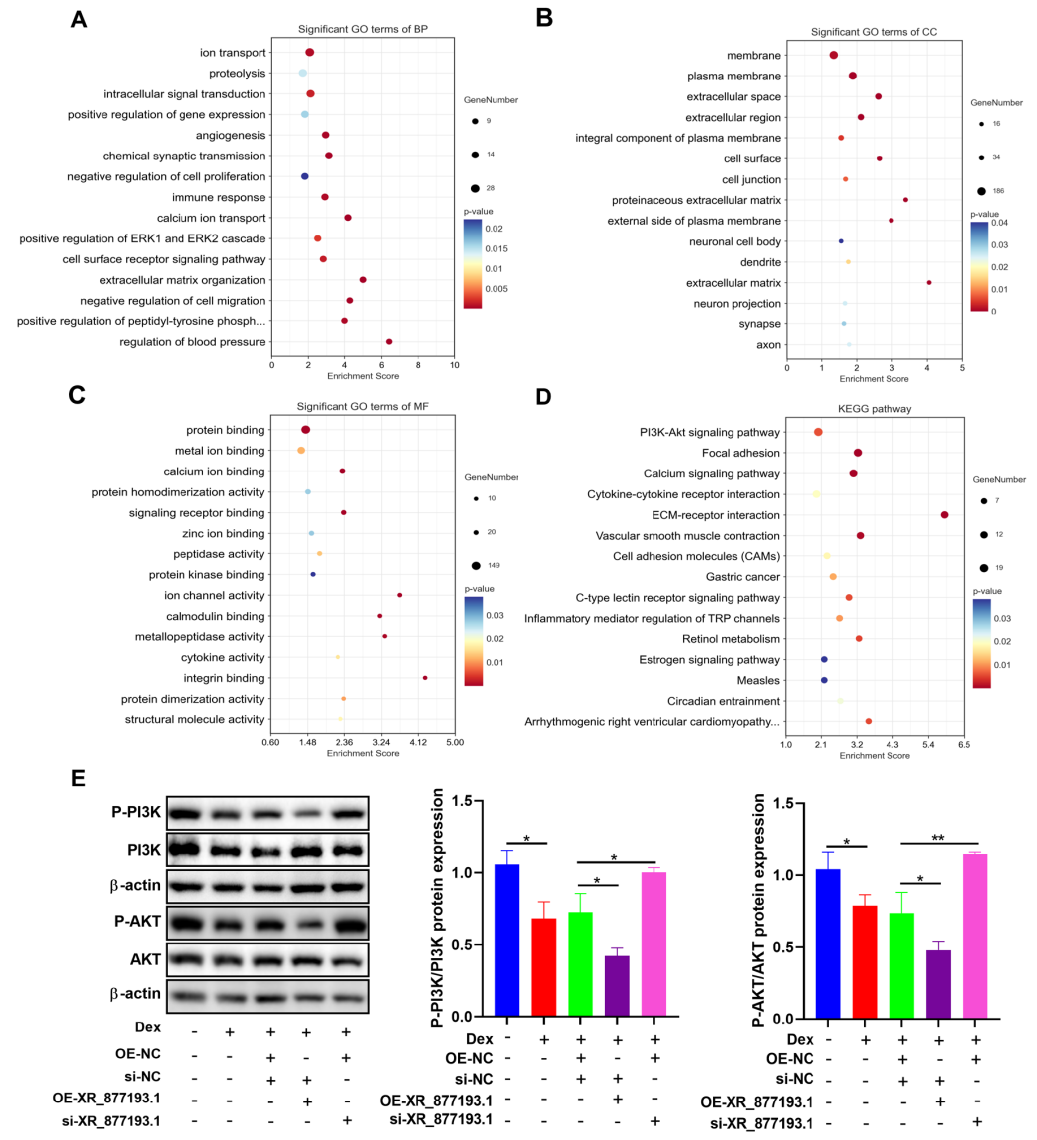
Figure 6 LncRNA XR_877193.1 suppresses PI3K/AKT signaling pathway in MC3T3-E1 cells (A–C) Top 15 enriched biological processes (BP), cellular components (CC), and molecular functions (MF) of differentially expressed lncRNA XR_877193.1 were determined by GO analysis in MC3T3-E1 cells. (D) Classification map of KEGG pathway enrichment analysis for differentially expressed lncRNA XR_877193.1 in MC3T3-E1 cells. (E) The expressions of phosphatidylinositol 3-kinase (PI3K), phospho-PI3K (P-PI3K), protein kinase B (AKT) and phospho-AKT (P-AKT) were detected by western blot analysis in MC3T3-E1 cells transfected with lncRNA XR_877193.1 overexpression plasmid or lncRNA XR_877193.1 siRNA in the presence of Dex (n = 3). Data are presented as the mean ± SD. *P < 0.05, ***P < 0.001.

To further elucidate the role of lncRNA XR_877193.1 in Dex-treated MC3T3-E1 cells, we performed GO and KEGG analyses on the target genes of the differentially expressed lncRNA XR_877193.1. The GO analysis focused primarily on biological processes (BP), molecular functions (MF), and cellular components (CC). BP was significantly enriched for ion transport, proteolysis, and intracellular signal transduction. Significant CC terms were enriched in the membrane, plasma membrane, and extracellular space. The top three MFs identified were protein binding, metal ion binding, and calcium ion binding. These enriched terms confirmed that aberrant lncRNA expression plays a crucial role in the pathogenesis and development of SONFH ( Figure 6A–C). KEGG analysis was subsequently performed to identify the main biochemical metabolic pathways and signal transduction pathways affected by the lncRNA XR_877193.1 target genes. The results indicated that several signaling pathways were abnormal, among which the PI3K/AKT signaling pathway had the largest number of differentially expressed genes ( Figure 6D). These findings suggest that the PI3K/AKT signaling pathway might be involved in the ferroptosis of MC3T3-E1 cells in SONFH. Thus, we measured PI3K and AKT activation in MC3T3-E1 cells following the overexpression of lncRNA XR_877193.1 or transfection with lncRNA XR_877193.1 siRNA. We observed significant decreases in PI3K and AKT phosphorylation in Dex-treated MC3T3-E1 cells overexpressing lncRNA XR_877193.1, whereas their phosphorylation levels increased in Dex-treated MC3T3-E1 cells transfected with lncRNA XR_877193.1 siRNA ( Figure 6E). Taken together, these results further indicate that lncRNA XR_877193.1 suppresses the PI3K/AKT signaling pathway in Dex-treated MC3T3-E1 cells.

### Knockdown of lncRNA XR_877193.1 inhibits Dex-induced ferroptosis via promoting the PI3K/AKT signaling pathway in MC3T3-E1 cells

**Figure 7:**
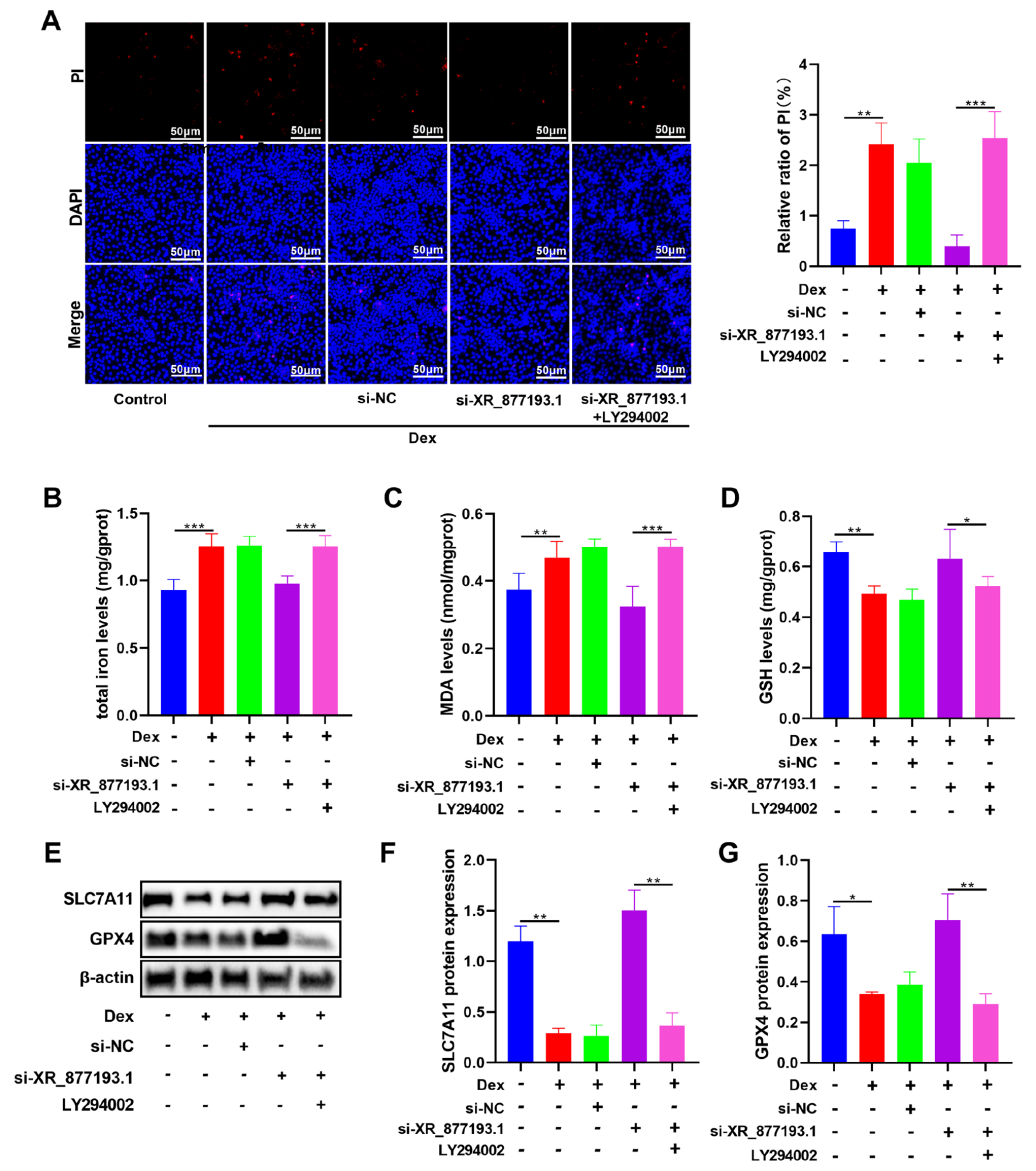
Figure 7 Knockdown of LncRNA XR_877193.1 inhibits Dex-induced ferroptosis by promoting PI3K/AKT signaling pathway in MC3T3-E1 cells (A) Representative images of PI (red) in si-XR_877193.1 and/or LY294002 MC3T3-E1 cells stimulated by Dex (n = 3). The nuclei were stained with DAPI (blue). Scale bar: 50 μm. (B–D) The concentrations of total iron, MDA and GSH were detected using biochemical kits in MC3T3-E1 cells transfected with lncRNA XR_877193.1 siRNA and/or LY294002 in the presence of Dex (n = 6). (E–G) The expressions of SLC7A11 and GPX4 were detected by western blot analysis in MC3T3-E1 cells transfected with lncRNA XR_877193.1 siRNA and/or LY294002 in the presence of Dex (n = 3). Data are presented as the mean ± SD. *P < 0.05, **P < 0.01, ***P < 0.001.

Given that lncRNA XR_877193.1 could regulate the PI3K/AKT signaling pathway in Dex-treated MC3T3-E1 cells, we hypothesized that lncRNA XR_877193.1 might also control ferroptosis in MC3T3-E1 cells via this pathway. To test this hypothesis, we used a specific inhibitor of the PI3K/AKT signaling pathway, LY294002, to determine whether the PI3K/AKT signaling pathway is involved in lncRNA XR_877193.1-regulated ferroptosis in MC3T3-E1 cells. We treated MC3T3-E1 cells with 40 μM LY294002 (Supplementary Figure S1). The results of PI staining demonstrated that LY294002 restored ferroptosis in Dex-treated MC3T3-E1 cells transfected with lncRNA XR_877193.1 siRNA ( Figure 7A). Further biochemical analyses revealed that LY294002 significantly weakened the regulatory effect of lncRNA XR_877193.1, notably increasing the levels of total iron and MDA but decreasing the level of GSH in Dex-treated MC3T3-E1 cells ( Figure 7B–D). Additionally, LY294002 reduced the levels of GPX4 and SLC7A11 in MC3T3-E1 cells ( Figure 7E–G). These results suggest that the knockdown of lncRNA XR_877193.1 could decrease ferroptosis in Dex-treated MC3T3-E1 cells by activating the PI3K/AKT signaling pathway.

## Discussion

SONFH is a systemic form of osteonecrosis caused by the massive use of glucocorticoids. The main symptoms of SONFH are hip pain, joint stiffness, and limited mobility. If there is no timely intervention, SONFH can cause the collapse of the articular surface and osteoarthritis and eventually lead to disability and deformity in patients [19]. Although the etiology, pathogenesis, diagnosis, and treatment of SONFH have been studied extensively, there is no clear consensus as to its exact origin. Osteoblasts are essential for synthesizing and depositing new bone tissue [20]. The impaired function of osteoblasts can result in decreased bone formation, compromised bone structure and increased susceptibility to bone damage [ 21, 22]. Research has shown that high-dose and long-term steroid treatment alters the antioxidative ability and decreases the viability and function of osteoblasts, leading to osteoporosis and osteonecrosis [23]. Several studies have also indicated that osteoblast ferroptosis and oxidative stress contribute to the pathological process of SONFH [19].

Ferroptosis is caused by the inhibition of GPX4 and SLC7A11, which leads to inhibition of cystine metabolism, enhanced lipid peroxidation, and iron metabolism dysfunction and causes the generation of a large amount of ROS, triggering cell death [24]. In the present study, we confirmed that Dex led to downregulation of the expression of GPX4 and SLC7A11 in MC3T3-E1 cells. Moreover, Dex increased total iron and MDA levels, decreased intracellular GSH levels, and caused mitochondria to shrink, whereas the number of mitochondrial ridges decreased significantly compared with those in the control group. However, Fer-1 reversed these changes and enhanced the expressions of the osteogenic markers OPN, Runx2, and OCN in Dex-treated cells. Thus, Fer-1 alleviates impaired osteogenic differentiation by inhibiting Dex-induced ferroptosis.

Long noncoding RNAs (lncRNAs) are regulatory RNA molecules that do not encode proteins but are crucial for gene expression, cell differentiation, and cell death [25]. They can influence ferroptosis by modulating the expressions of antioxidant genes, such as *GPX4*, and regulating iron metabolism-related proteins, thereby affecting intracellular iron levels and cellular sensitivity to ferroptosis [26]. Lin *et al*. [27] reported that hypoxia-induced HIF-1α/lncRNA-PMAN inhibits ferroptosis by promoting the cytoplasmic translocation of ELAVL1 during peritoneal dissemination from gastric cancer. However, the function and molecular mechanism of lncRNAs in SONFH ferroptosis remain to be clarified. In this study, lncRNA microarray analysis of MC3T3-E1 cells revealed significant dysregulation of lncRNAs, with 254 upregulated and 138 downregulated in Dex-induced cells, suggesting the involvement of lncRNAs in SONFH. Notably, lncRNA XR_877193.1 was significantly upregulated in Dex-treated MC3T3-E1 cells and potentially linked to Dex-induced ferroptosis. Further studies revealed that the overexpression of lncRNA XR_877193.1 decreased calcified nodule formation and the expressions of osteogenic markers, increased ROS levels, and reduced GPX4 and SLC7A11 levels. Conversely, knockdown of lncRNA XR_877193.1 increased calcified nodule formation and the expressions of osteogenic markers, decreased ROS levels, and increased GPX4 and SLC7A11 expression. Taken together, these results indicated that lncRNA XR_877193.1 inhibited Dex-induced osteogenic differentiation and facilitated ferroptosis in MC3T3-E1 cells.

LncRNAs participate in several aspects of SONFH and regulate the expressions of key components of related pathways [ 28, 29]. In the present study, we found that the lncRNA XR_877193.1 is a mediator of MC3T3-E1 cell ferroptosis. LncRNAs can act by activating specific signaling pathways, which is one of the mechanisms involved [30]. For example, LINC01121 inhibits cell apoptosis while facilitating proliferation, migration, and invasion though negative regulation of the Camp/PKA signaling pathway via GLP1R [31]. LINC02159 promotes non-small cell lung cancer progression via ALYREF/YAP1 signaling [32]. Here, GO annotation and KEGG enrichment analysis of the differentially expressed lncRNA XR_877193.1 were performed. The PI3K/AKT signaling pathway had the highest enrichment, suggesting that this signaling pathway may play important roles in SONFH. Moreover, we found that downregulation of lncRNA XR_877193.1 expression activated the PI3K/AKT signaling pathway. The PI3K/AKT signaling pathway controls many cellular processes, including cell division, autophagy, survival, and differentiation [33]. This could explain why lncRNA XR_877193.1 knockdown suppressed MC3T3-E1 cell ferroptosis. By using a specific inhibitor of the signaling pathway, we further confirmed that lncRNA XR_877193.1 regulated MC3T3-E1 cell ferroptosis through the PI3K/AKT signaling pathway.

In summary, we found that lncRNA XR_877193.1, a significantly upregulated lncRNA under Dex treatment, facilitated Dex-induced ferroptosis in MC3T3-E1 cells and regulated MC3T3-E1 cell ferroptosis through the PI3K/AKT signaling pathway during the progression of SONFH. This study provides a new theoretical basis for SONFH progression and a possible approach for the targeted treatment of SONFH. Nevertheless, the limitation of this work is that only *in vitro* experiments were carried out, and the strong evidence from animal experiments will make this study more convincing and meaningful. Addressing this limitation may provide solid evidence for the pathogenesis of SONFH.

## Supplementary Data

Supplementary data is available at *Acta Biochimica et Biophysica Sinica* online.
